# Methyl‐Substituted α‐Cyclodextrin as Affinity Material for Storage, Separation, and Detection of Trichlorofluoromethane

**DOI:** 10.1002/gch2.201800057

**Published:** 2018-08-12

**Authors:** Dimitrij Ryvlin, Maiko Girschikofsky, Dieter Schollmeyer, Ralf Hellmann, Siegfried R. Waldvogel

**Affiliations:** ^1^ Institute of Organic Chemistry Johannes Gutenberg University Mainz Duesbergweg 10–14 D‐55128 Mainz Germany; ^2^ Applied Laser and Photonics Group University of Applied Sciences Aschaffenburg Würzburger Straße 45 D‐63743 Aschaffenburg Germany

**Keywords:** CFC‐11, cyclodextrin, optical sensors, supramolecular chemistry, trichlorofluoromethane

## Abstract

The severely ozone‐depleting trichlorofluoromethane is still appearing in several recycling processes or industrial applications. A simple and selective supramolecular complex formation of *per*‐methylated α‐cyclodextrin (**1**) with the highly volatile trichlorofluoromethane (**2**) is reported. This interaction moreover leads to thermally stable crystals. *Per*‐methylated α‐cyclodextrin is successfully exploited as a reversible and selective adsorption material for liquid and airborne trichlorofluoromethane as well as an affinity material for the chemical sensing and detection of this particular volatile organic component.

During the past century, chlorofluorocarbons (CFCs) were widely used as refrigerants, propellants, and solvents for various applications. However, due to their ozone depleting potential, the production and the utilization of CFCs were agreed upon to be phased out by the international community under the Montreal Protocol in 1987, with the result of a now steadily recovering ozone layer.^1^, ^2^ Until the complete phase‐out of CFCs in 1996 and 2010 for industrial and developing countries, respectively, a large amount of these environmentally harmful substances, such as trichlorofluoromethane (**2**) (referred to as CFC‐11, R‐11, Freon‐11, Arcton‐9, etc.) were accumulated in terms of either stockpiled or product‐bound CFCs and therefore, still leak into the atmosphere and impair the ozone layer.^2^, ^3^ Recently, Montzka et al. could show a re‐emerging and strong increase in the global CFC‐11 release.^4^ This setback demonstrates dramatic situation and manifests the contemporary nature of this hot topic. Therefore, a sufficient detection and emission‐free recovery of these accumulated substances became one of the central issues. Today's state‐of‐the‐art technologies for an adequate handling of CFCs typically rely on cryo‐condensation,^5^, ^6^ adsorption (particularly on activated carbon),^5^, ^7^ or incineration.^5^, ^8^ With these technologies, however, a selective extraction of defined CFCs such as CFC‐11 is either not possible or both sophisticated and expensive.^9^ Therefore, simple and selective approaches for binding CFC‐11 are highly desired.

A promising approach for a selective adsorption relies on molecular recognition, which is based on a noncovalent supramolecular complex formation of a guest molecule with a suitable host.^10^ Accordingly, Dichtel and co‐workers demonstrated the ability to encapsulate fluorinated pollutants using cyclodextrin,^11^ one of the most extensively studied and applied groups of supramolecular hosts. In general, cyclodextrins are cyclic oligosaccharides typically containing six, seven, or eight glucopyranose units which are referred to as α‐, β‐, and γ‐cyclodextrins, respectively.^12^, ^13^, ^14^ These toroid‐shaped cyclic oligosaccharides feature a hydrophobic interior and a hydrophilic exterior, which enable cyclodextrins to form noncovalent but solid host–guest inclusion complexes.^12^, ^14^, ^15^, ^16^ Further characteristics such as the solubility, the viscosity, and the complex‐forming selectivity are almost arbitrarily modifiable by an appropriate substitution of the cyclodextrins' hydroxyl groups.^12^, ^13^, ^17^ As a consequence, over the past decades, numerous cyclodextrin derivatives of manifold chemical and physical properties have been synthesized, studied, and by now led to a broad variety of commercially available, inexpensive, and easily accessible products.^12^, ^13^, ^16^, ^18^ Therefore, cyclodextrins could represent a suitable supramolecular host for the complexation of **2**. In particular, due to its dimension, the cyclodextrin derivative hexakis(2,3,6‐tri‐*O*‐methyl)‐α‐cyclodextrin (**1**) provides an ideal cavity size for the complexation of **2** (**Figure** **1**).

**Figure 1:**
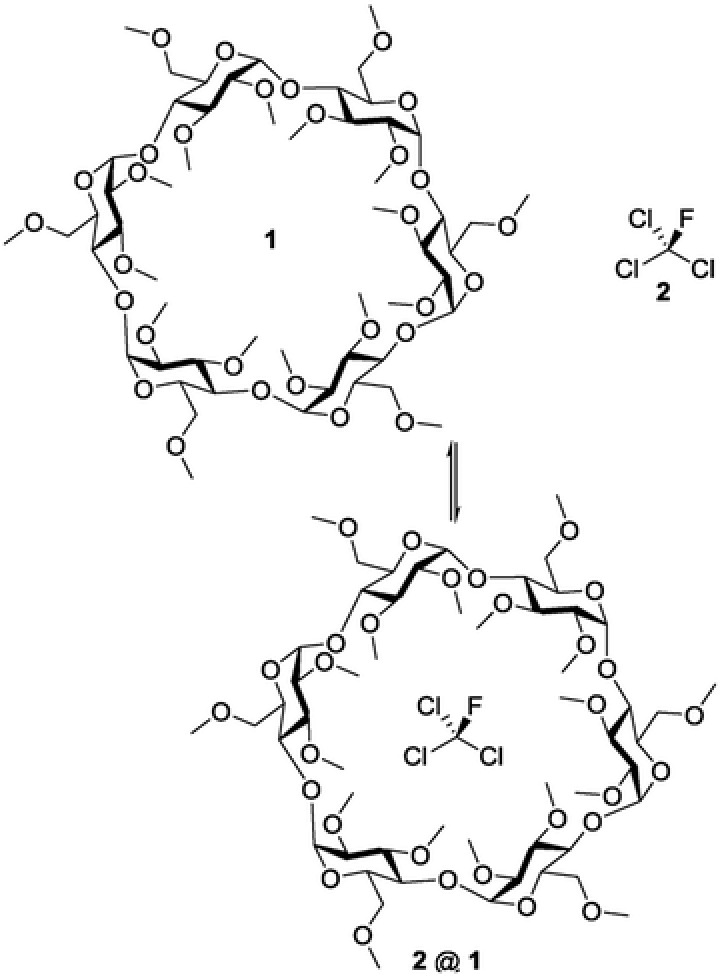
Hexakis(2,3,6‐tri‐*O*‐methyl)‐α‐cyclodextrin, trichlorofluoromethane, and the proposed complex (**2** @ **1**).

To confirm this hypothesis, hexakis(2,3,6‐tri‐*O*‐methyl)‐α‐cyclodextrin was prepared in larger quantity. The synthesis of *per*‐methylated α‐cyclodextrin is well known.^19^ However, here a synthetic protocol is established that allows the use of caustic soda as base and methyl *p*‐toluenesulfonate as methylation agent and so enables a synthesis of **1** on multigram scale in a safe, inexpensive, and practical way (see Supporting Information).^20^

The easy formation of the complex (**2** @ **1**) is demonstrated when **1** as powder is directly treated with liquid **2**. Due to the excellent complementary size of **2** with the cyclodextrin cavity, **2** forms a stable supramolecular complex as clear colorless crystals within less than 1 h. The spatial arrangement was elucidated by X‐ray diffraction analysis of suitable single crystals. The X‐ray analysis of the stable crystals confirms the supramolecular complexation of **2** in the lattice of the *per*‐methylated α‐cyclodextrin, wherein four trichlorofluoromethane molecules are enclosed per **1** (**Figure** **2**). Thereby, two of the guests are positioned in the interstitial positions of the adjacent cyclodextrins, one is halfway inside the cyclodextrin cavity at its primary side and the remaining guest is located fully inside the cyclodextrin. More details about the X‐ray analysis can be found in Supporting Information.

**Figure 2:**
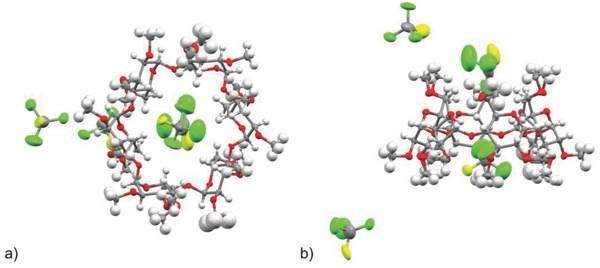
Thermal ellipsoid molecular structure of the crystallized **2** @ **1** determined by X‐ray crystal structure analysis. a) Top view onto the cyclodextrin's secondary side. b) Side view. Color code: C gray, O red, Cl green, F yellow, H white.

Thermogravimetric analysis (TGA) of the crystallized supramolecular complex reveals a thermal stability of **2** @ **1** up to a temperature of 85 °C (**Figure** **3**). This temperature marks the onset of a stepwise release of **2** and is more than 60 °C higher than the boiling point of **2** (b.p. 23.7 °C).^20^, ^21^ Up to 195 °C, a weight loss of ≈28 wt% can be found, which confirms the 4:1 complex ratio of **2** and **1**. These findings are in good accordance with the theoretically achievable 30 wt%. Further TGA‐MS analysis reveals that exclusively **2** is released, whereas the cyclodextrin is thermally stable up to a temperature of 270 °C. In order to investigate the long‐term stability of the crystallized supramolecular complex at ambient conditions, an additional TGA measurement is performed with the crystals being exposed to a constant nitrogen flow of 20 mL·min^−1^ at isothermal conditions (25 °C) over a period of 12 h. During this treatment, a weight loss of only 3 wt% is observed, which proves the good stability of the complex.

**Figure 3:**
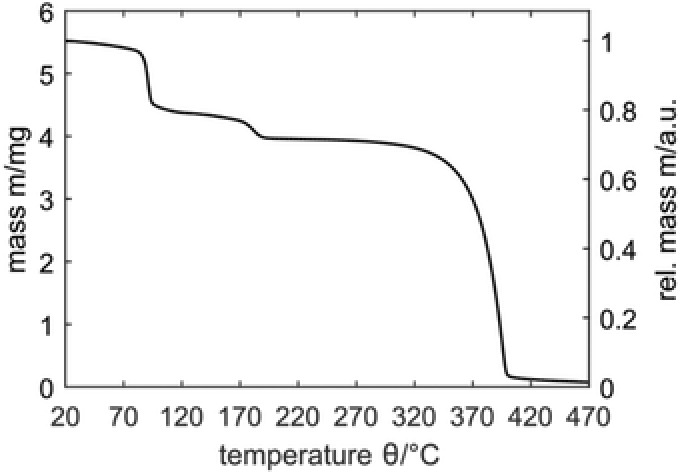
Thermal gravimetric analysis of **2** @ **1** at a heat‐rate of 5 K∙min^−1^.

Based on the investigated reversibility and selectivity of the adsorption of **2** by **1**, this particular cyclodextrin‐derivative can be expected to be a promising adsorption material for airborne **2**, which, e.g., occurs in the recycling process of waste refrigerators.^5^

Accordingly, **1** is applied as an affinity material for packed‐bed adsorption‐tubes. Therefore, a 14.7 mL glass tube is packed with 6.45 g of the powdery *per*‐methylated α‐cyclodextrin and exposed to a **2** containing (50 vol%) nitrogen stream. Here, an average weight increase of the packed‐bed adsorption‐tube of 20% can be achieved (see Supporting Information). The stability of the complexation at ambient conditions is investigated by purging the saturated packed‐bed adsorption‐tube with a constant nitrogen flow over a period of 12 h resulting in a weight decrease of an average of 5%. Moreover, by heating the adsorption‐tube to a temperature above 85 °C for 12 h while purging with pure nitrogen, the adsorbed **2** can completely be recovered. Subsequently, this adsorption‐tube could be reused to adsorb **2** out of the gas stream multiple times with the same performance.

The selectivity of **1** to form a supramolecular complex with **2** is investigated by exposition of the *per*‐methylated α‐cyclodextrin to an unbalanced binary mixture of **2** and dichloromethane (DCM) at the ratio of 5:95 v/v. Since DCM has comparable dimensions to **2**, the statistical distribution of **2** and DCM in the complexation by **1** should be proportional to the ratio of the binary mixture. The complexation of **2** and DCM by *per*‐methylated α‐cyclodextrin as well results in a crystallization. However, despite **2** having only a volume fraction of 5%, the X‐ray analysis of the crystallized supramolecular complex reveals an invariable occupation of the *per*‐methylated α‐cyclodextrin's cavity by **2** and is thus 20 times higher than statistically expected, while three further positions within the crystal lattice are occupied by DCM molecules (see Supporting Information).

Due to the investigated properties, the application of this cyclodextrin‐derivative allows an easily manageable adsorption, retention, and desorption of the ozone‐depleting **2** with no need of further precautions such as cryogenic equipment or the employment of septum‐sealed bottles.

The ability of **1** to adsorb and efficiently retain gaseous **2** with the option of a temperature‐triggered desorption paves the way for the fabrication of low‐cost, reusable, and therefore highly sustainable filter elements for the environmentally harmful trichlorofluoromethane.

Furthermore, these properties provide the base for an application of **1** as an affinity material in the reversible sensing of airborne **2**. For this purpose, an optical planar Bragg grating sensor is applied, which is based on a monochromatic reflective element within an optical waveguide. Planar Bragg grating sensors represent compact and lightweight, but robust refractive index sensors that feature a very low signal attenuation, immunity to electromagnetic interferences, and multiplexing capability (see Supporting Information).^22^ The wavelength that is reflected by the Bragg grating, referred to as the Bragg wavelength (λ_B_), is, amongst others, depending on the Bragg grating's immediate vicinity, whereby changes of the surrounding medium's refractive index lead to a detectable and traceable shift of λ_B_. For the sensitization of the planar Bragg grating sensor to **2**, the *per*‐methylated α‐cyclodextrin is applied as a thin layer of ≈150 nm onto the sensitive surface area of the sensor (see Supporting Information). By this measure, the planar Bragg grating sensor exhibits a rapid and reversible response, whereby the overall signal deflection follows a Langmuir–Freundlich isotherm (*R* ^2^ = 0.999) on the concentration of the applied **2**.^23^ Taking into account the resolution and background noise of the applied interrogation system, the sensory response found here deduces a limit of detection for **2** of less than 100 ppm. The efficiency of the *per*‐methylated α‐cyclodextrin based functionalization is apparent as soon as the sensitized sensor's response is compared to that of an identical but unmodified sensor. For an exemplary **2** concentration of 10 vol%, the unmodified sensor exhibits only a negligible signal deflection of ≈1.8 pm, whereas the sensor coated with **1** features a Bragg wavelength shift of ≈82 pm which represents a 45‐fold signal increase (**Figure** **4**a).

**Figure 4:**
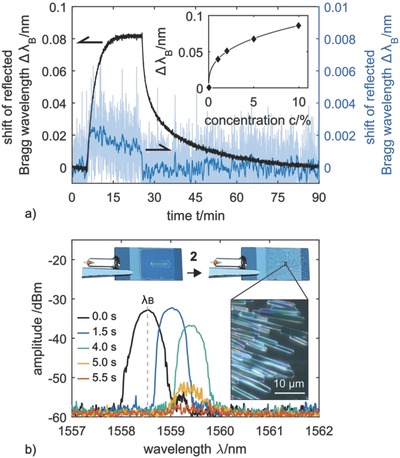
a) Signal response (Δλ_B_) of a planar Bragg grating sensor coated with **1** (solid black line) and an identical but uncoated sensor (solid blue line) to a **2** exposition of 10 vol% in nitrogen. The inset shows the signal deflection of the coated sensor to various concentrations of **2** and the corresponding Langmuir–Freundlich fit. b) Shift of the reflected Bragg wavelength with signal drop‐off, due to crystallization. The images show the coated planar Bragg grating sensor prior and after exposure to **2**. The microscopic image shows the crystallized sensor surface.

Remarkably, for CFC‐11 concentrations above 35 vol% a spontaneous, clearly visible crystallization of the amorphous cyclodextrin sensitization layer occurs within only a few seconds. This crystallization leads to an instantaneous signal drop‐off of the sensor (Figure 4b), which can be attributed to two reasons. Firstly, an increasing refractive index of the cyclodextrin layer due to the deposition of **2**, resulting in a cumulative pull of the guided mode toward the surface of the optical waveguide. Secondly, to scattering losses by crystallization‐caused structural discontinuity of the sensor surface. This behavior of crystallization with a drop‐off of the sensor signal can easily be adapted as a one‐way sensor, e.g., for an on‐site real‐time surveillance of industrial production plants or end‐of‐life refrigerator treatment facilities, where **2** might be released and can leak into the atmosphere.

Further details about the sensor, its sensitization with **1**, and the experimental setup can be found in Supporting Information.

In this work, we demonstrated the ability of hexakis(2,3,6‐tri‐*O*‐methyl)‐α‐cyclodextrin to selectively form a stable supramolecular complex with trichlorofluoromethane. The reversible complexation of **2** with **1** results in a highly efficient crystallization at a ratio of 4:1. Furthermore, we demonstrated the applicability of hexakis(2,3,6‐tri‐*O*‐methyl)‐α‐cyclodextrin as a packing material for packed‐bed adsorption‐tubes and thus might facilitate the fabrication of economically reusable and highly sustainable filter elements for trichlorofluoromethane.

Moreover, an application of **1** as an affinity material in the chemical sensing of airborne CFC‐11 was accomplished using an opto‐chemical planar Bragg grating sensor that exhibited a profound, rapid, and reversible signal deflection to **2** exposition that is 45‐fold higher as compared to a nonsensitized sensor. Additionally, CFC‐11 concentrations above 35 vol% lead to visible spontaneous crystallization of the sensor's sensitization coating and an immediate signal drop‐off, which can easily be adapted as a one‐way sensor, i.e., for surveillance of industrial production plants or end‐of‐life refrigerator treatment facilities.

## Conflict of Interest

The authors declare no conflict of interest.

## Supporting information

Supplementary

## References

[1] J. A. Mäder , J. Staehelin , T. Peter , D. Brunner , H. E. Rieder , W. A. Stahel , Atmos. Chem. Phys. 2010, 10, 12161.

[2] United Nations Environment Programme , Ozone Secretariat, Handbook for the Montreal Protocol on Substances that Deplete the Ozone Layer, 10th Ed., Nairobi, Kenya 2016.

[3] B. Yazici , Z. S. Can , B. Calli , Waste Manage. 2014, 34, 162. 10.1016/j.wasman.2013.09.008 24112854

[4] S. A. Montzka , G. S. Dutton , P. Yu , E. Ray , R. W. Portmann , J. S. Daniel , L. Kuijpers , B. D. Hall , D. Mondeel , C. Siso , J. D. Nance , M. Rigby , A. J. Manning , L. Hu , F. Moore , B. R. Miller , J. W. Elkins , Nature 2018, 557, 413. 29769666 10.1038/s41586-018-0106-2

[5] J. Ruan , Z. Xu , Waste Manage. 2011, 31, 2319. 10.1016/j.wasman.2011.06.004 21782408

[6] D. Laner , H. Rechberger , Resour., Conserv. Recycl. 2007, 52, 136.

[7] a) C. Mellot‐Draznieks , J. Rodriguez‐Carvajal , D. E. Cox , A. K. Cheetham , Phys. Chem. Chem. Phys. 2003, 5, 1882;

[8] a) M. Y. Hiraiwa , A. Yamazaki , T. Nagoya , J. Ceram. Soc. Jpn. 1996, 104, 264;

[9] S. Ordóñez , M. Makkee , J. A. Moulijn , Appl. Catal., B 2001, 29, 13.

[10] a) J. W. Steed , J. L. Atwood , Supramolecular Chemistry, Wiley, Chichester, UK 2009;

[11] D. M. Alzate‐Sánchez , B. J. Smith , A. Alsbaiee , J. P. Hinestroza , W. R. Dichtel , Chem. Mater. 2016, 28, 8340.

[12] H. Dodziuk (Ed.), Cyclodextrins and Their Complexes: Chemistry, Analytical Methods, Applications, Wiley‐VCH, Weinheim 2006.

[13] E. M. M. Del Valle , Process Biochem. 2004, 39, 1033.

[14] J. Szejtli , Chem. Rev. 1998, 98, 1743.11848947

[15] W. Saenger , J. Jacob , K. Gessler , T. Steiner , D. Hoffmann , H. Sanbe , K. Koizumi , S. M. Smith , T. Takaha , Chem. Rev. 1998, 98, 1787. 11848949 10.1021/cr9700181

[16] S. D. Eastburn , B. Y. Tao , Biotechnol. Adv. 1994, 12, 325. 14545896 10.1016/0734-9750(94)90015-9

[17] J. Szejtli , J. Inclusion Phenom. Mol. Recognit. Chem. 1992, 14, 25.

[18] M. T. Reetz , S. R. Waldvogel , Angew. Chem. 1997, 109, 870;

[19] a) Y. Akae , Y. Koyama , H. Sogawa , Y. Hayashi , S. Kawauchi , S. Kuwata , T. Takata , Chem. ‐ Eur. J. 2016, 22, 5335; 26914705 10.1002/chem.201504882

[20] D. R. Lide , CRC Handbook of Chemistry and Physics: A Ready‐Reference Book of Chemical and Physical Data, CRC Press, Boca Raton, FL 1994.

[21] a) R. Schmidt , H. D. Brauer , J. Am. Chem. Soc. 1987, 109, 6976;

[22] a) Y. J. Rao , Opt. Lasers Eng. 1999, 31, 297;

[23] a) O. Redlich , D. L. Peterson , J. Phys. Chem. 1959, 63, 1024;

