# Supplementary material for: Methyl‐Substituted α‐Cyclodextrin as Affinity Material for Storage, Separation, and Detection of Trichlorofluoromethane
Source: Glob Chall. 2018 Aug 12;2(8):1800057. doi: 10.1002/gch2.201800057 (PMC6607158; doi:10.1002/gch2.201800057)
Supplement: Supplementary file 1 — Supplementary [file GCH2-2-1800057-s001.pdf]

Copyright WILEY-VCH Verlag GmbH & Co. KGaA, 69469 Weinheim, Germany, 2018.

Global Challenges Open Access

Supporting Information

for *Global Challenges,* DOI: 10.1002/gch2.201800057

Methyl-Substituted α-Cyclodextrin as Affinity Material for Storage, Separation, and Detection of Trichlorofluoromethane

Dimitrij Ryvlin, Maiko Girschikofsky, Dieter Schollmeyer, Ralf Hellmann, and Siegfried R. Waldvogel**

WILEY-VCH

Copyright WILEY-VCH Verlag GmbH & Co. KGaA, 69469 Weinheim, Germany, 2016.

Supporting Information

Methyl-Substituted α-Cyclodextrin as Affinity Material for Storage, Separation, and Detection of Trichlorofluoromethane

Dimitrij Ryvlin,[†] Maiko Girschikofsky,[†] Dieter Schollmeyer, Ralf Hellmann, and Siegfried R. Waldvogel**

S1. General remarks 3
S2. Synthesis of hexakis(2,3,6-tri-*O*-methyl)-α-cyclodextrin (1) 4
S3. X-Ray crystal structure of hexakis(2,3,6-tri-*O*-methyl)-α-cyclodextrin crystallized from trichlorofluoromethane 7
S4. X-Ray crystal Structure of hexakis(2,3,6-tri-*O*-methyl)-α-cyclodextrin crystallized from a mixture of dichloromethane and trichlorofluoromethane (95:5) 10
S5. TGA and TGA-MS experiments of hexakis(2,3,6-tri-*O*-methyl)-α-cyclodextrin inclusion complex with trichlorofluoromethane (CFC-11) 12
S6. Gas mixing unit 13
S7. Adsorption of airborne CFC-11 by hexakis(2,3,6-tri-*O*-methyl)-α-cyclodextrin 14
S8. Fabrication and principle of the planar Bragg grating sensor 15
S9. PBG sensor coating and layer thickness determination 18
S10. Detection of gaseous CFC-11 using a hexakis(2,3,6-tri-*O*-methyl)-α-cyclodextrin coated planar Bragg grating 18
S11. Crystallization of the surface coating on the Bragg grating sensor 19
S12. References 21

S1. General remarks

The reagents applied in the here presented work are commercially available and are purchased from Acros Organics, Fluka, Sigma-Aldrich, and Wacker-Chemie GmbH as reagent-grade without further purification.
For thin-layer chromatography, silica gel 60 sheets (F^{254}, Merck, Darmstadt, Germany) are applied.
^{1}H- and ^{13}C-NMR spectra are recorded using a Bruker AVANCE II 400 NMR (Analytische Messtechnik, Karlsruhe, Germany). ^{1}H NMR experiments are reported in δ units as parts per million (ppm) and are referenced to the signal for residual d_1-chloroform (7.26 ppm) in the solvent. ^{13}C-NMR spectra are reported in ppm relative to deuterated chloroform (77.16 ppm) and are obtained by ^{1}H decoupling.
IR spectra are recorded using an ALPHA FTIR with Platinum ATR module (Bruker, Billerica, Massachusetts, USA) running OPUS 7.5 software.
Exact mass determination was carried out on a 6545 Q-ToF (Agilent GmbH, Waldbronn) using Electrospray Ionization (ESI), sample loop inlet and an injection volume of 1 µL. Mass calibration was carried out on the day of analysis using an external standard mixture.
X-ray analyses are performed on a STOE IPDS2T diffractometer (Oxford Cryostream 700er series, Oxford Cryosystems) using graphite monochromated Mo-Kα radiation (λ = 0.71073 Å) and Incoatec microSource Cu-Kα radiation (λ = 1.54186 Å). Intensities are measured using fine-slicing ω and φ-scans and corrected with respect to background-, polarization-, and Lorentz effects. The determined structures are solved by direct methods and refined anisotropically by the least-squares procedure implemented in the SHELX program system.[S1] CCDC-1841752 and CCDC-1841753 contain the supplementary crystallographic data for this paper. These data can be obtained free of charge from The Cambridge Crystallographic Data Centre, 12 Union Road, Cambridge CB2 1EZ, UK (fax: +44(1223)-336-033; e-mail: deposit@ccdc.cam.ac.uk), or online from the website of The Cambridge Crystallographic Data Centre.
TGA-MS analyses are performed on a Mettler Toledo TGA / DSC1 Star°e System Thermobalance – Mass Spectrometry coupled with a Pfeiffer Thermostar TM apparatus. Further TGA analyses are performed on a Perkin Elmer Pyris 6 instrument, at a heat rate of 5 K/min and nitrogen as carrier gas with a gas flow of 20 mL/min.
The nitrogen used in the adsorption experiments features a purity of 99.998%.

S2. Synthesis of hexakis(2,3,6-tri-*O*-methyl)-α-cyclodextrin (1)

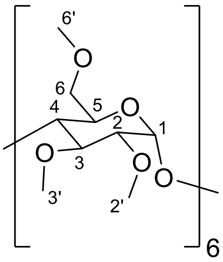

Finely powdered sodium hydroxide (24.00 g, 0.6 mol) is added to a solution of α-cyclodextrin (8.82 g, 9.1 mmol) in DMSO (270 mL) under vigorous mechanical stirring at room temperature until the mixture forms a slurry. Subsequently, the mixture is chilled to 0 °C and methyl *p*-toluenesulfonate (53 mL, 65.18 g, 0.35 mmol) is added dropwise under vigorous stirring and slowly warmed to ambient temperature. After stirring for 24 h, the reaction mixture is given onto ice-water-mixture (1 L). Afterwards, this mixture is extracted by dichloromethane (3 x 150 mL), the combined organic fractions are washed with brine (3 x 150 mL), dried over $MgSO_4$, filtered, and concentrated in vacuum. The crude product is crystallized from a mixture of acetone and cyclohexane (3:2) and dried in vacuum ($1 \cdot 10^{-3}$ mbar) at 80 °C in order to get hexakis(2,3,6-tri-*O*-methyl)-α-cyclodextrin as colorless solid in 93% yield (10.45 g, 8.5 mmol).

m.p.: 202-204 °C.

R_F (toluene:EtOH = 5:1) = 0.37.

^{1}H NMR (400 MHz, $CDCl_3$, 298 K): δ [ppm] = 3.16 (dd, 6H, *J* = 9.4 Hz, 3.2 Hz, 2-H), 3.40 (s, 18H, 6'-H), 3.48 (s, 18H, 2'-H), 3.54 (m, 12H, 4-H, 6a-H), 3.63 (s, 18H, 3'-H), 3.69 (d, 6H, *J* = 8.9 Hz, 3-H), 3.80 (m, 12H, 5-H, 6b-H), 5.05 (d, 6H, *J* = 3.2 Hz, 1-H).

^{13}C NMR (100 MHz, $CDCl_3$, 298 K): δ [ppm] = 57.9, 59.1, 61.9, 71.3, 71.5, 81.3, 82.3, 82.6, 100.2.

HRMS (ESI+): m/z calc. for $C_{54}H_{96}O_{30}Na$ $[M+Na]^+$: 1247.5879,

found : 1247.5887.

All analytical data match to previously reported data.[S2]

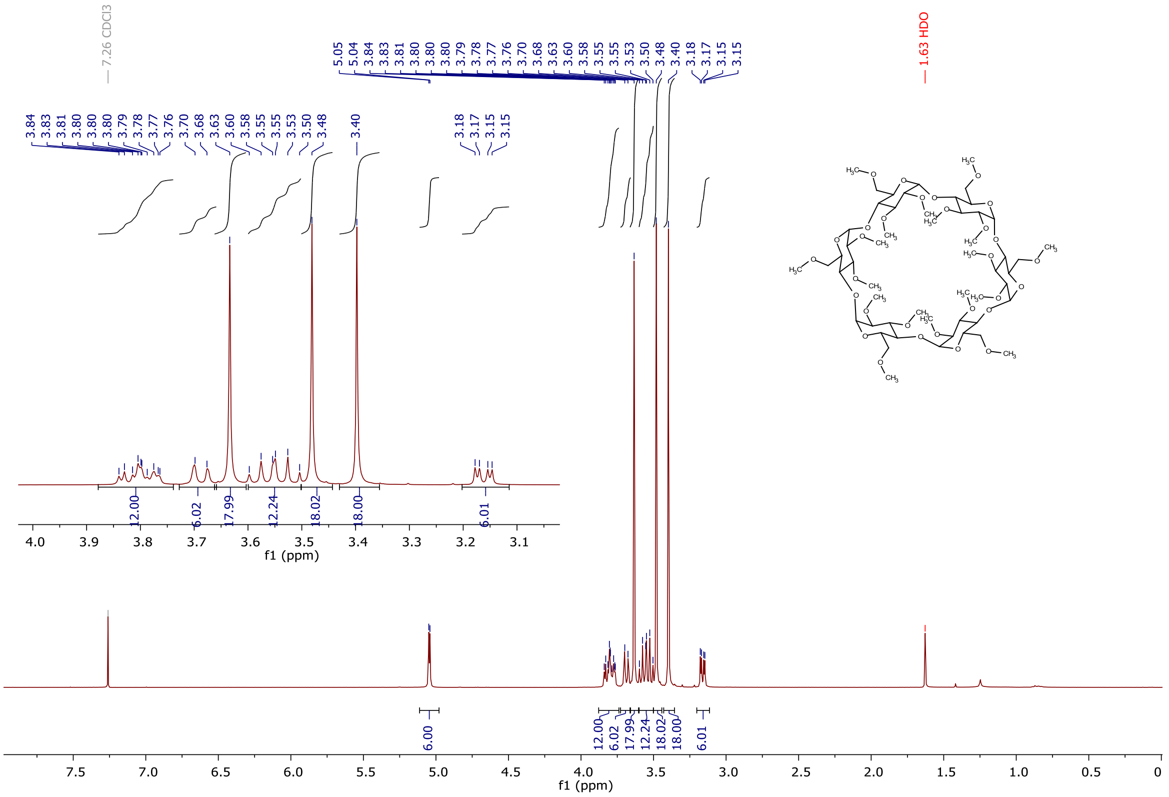

^{1}H NMR (400 MHz, CDCl$_3$)

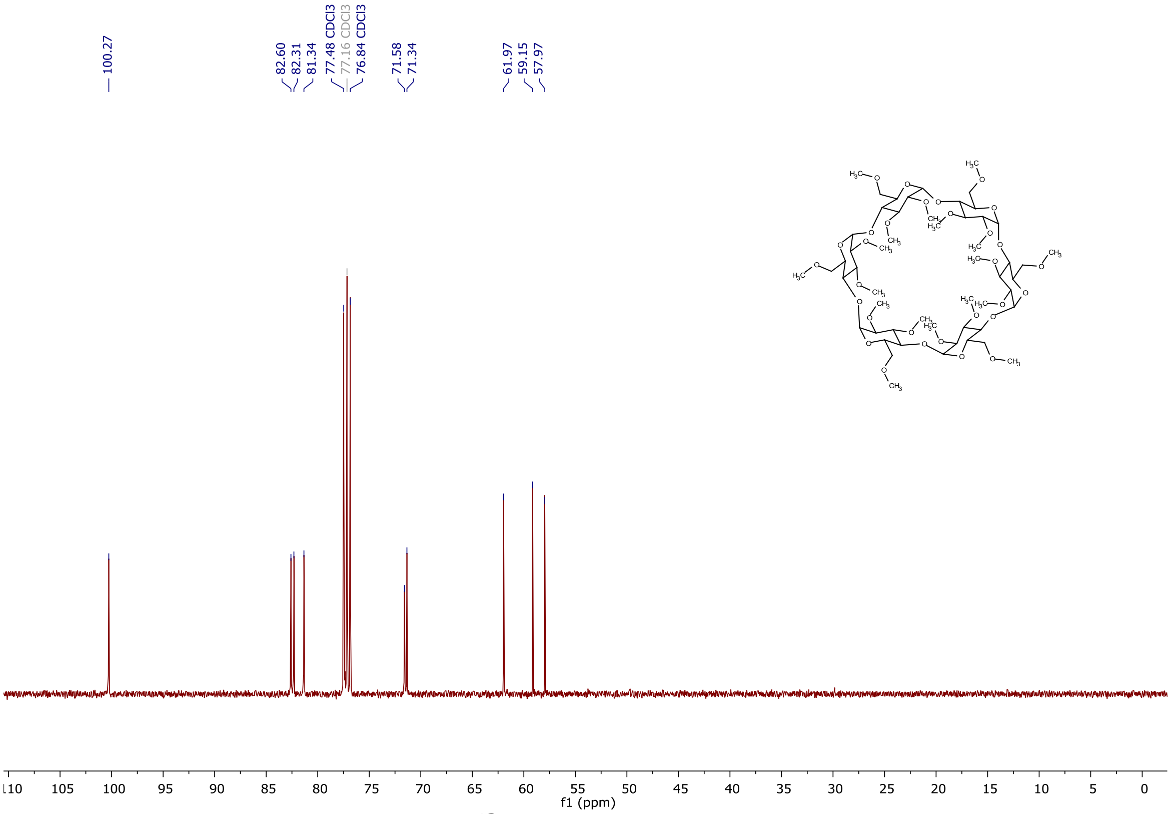

^{13}C NMR (101 MHz, CDCl$_3$)

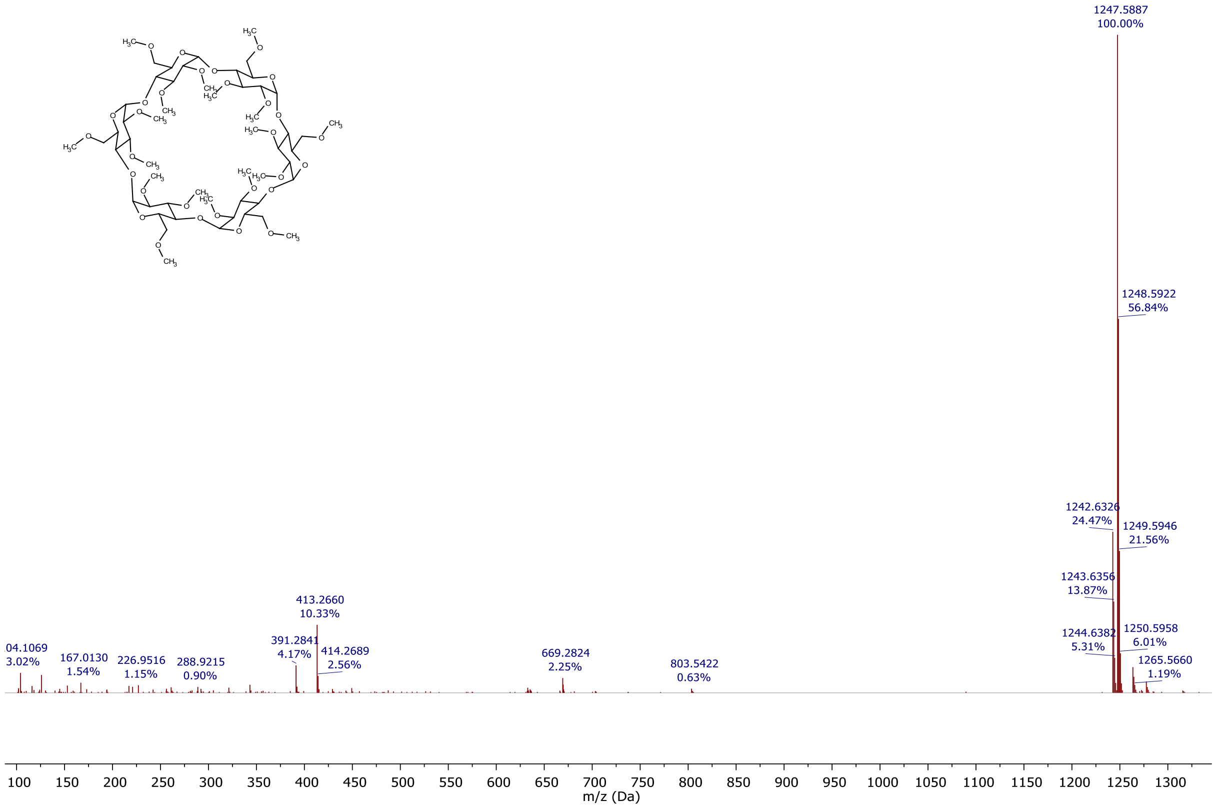

Mass spectra of hexakis(2,3,6-tri-*O*-methyl)-α-cyclodextrin.

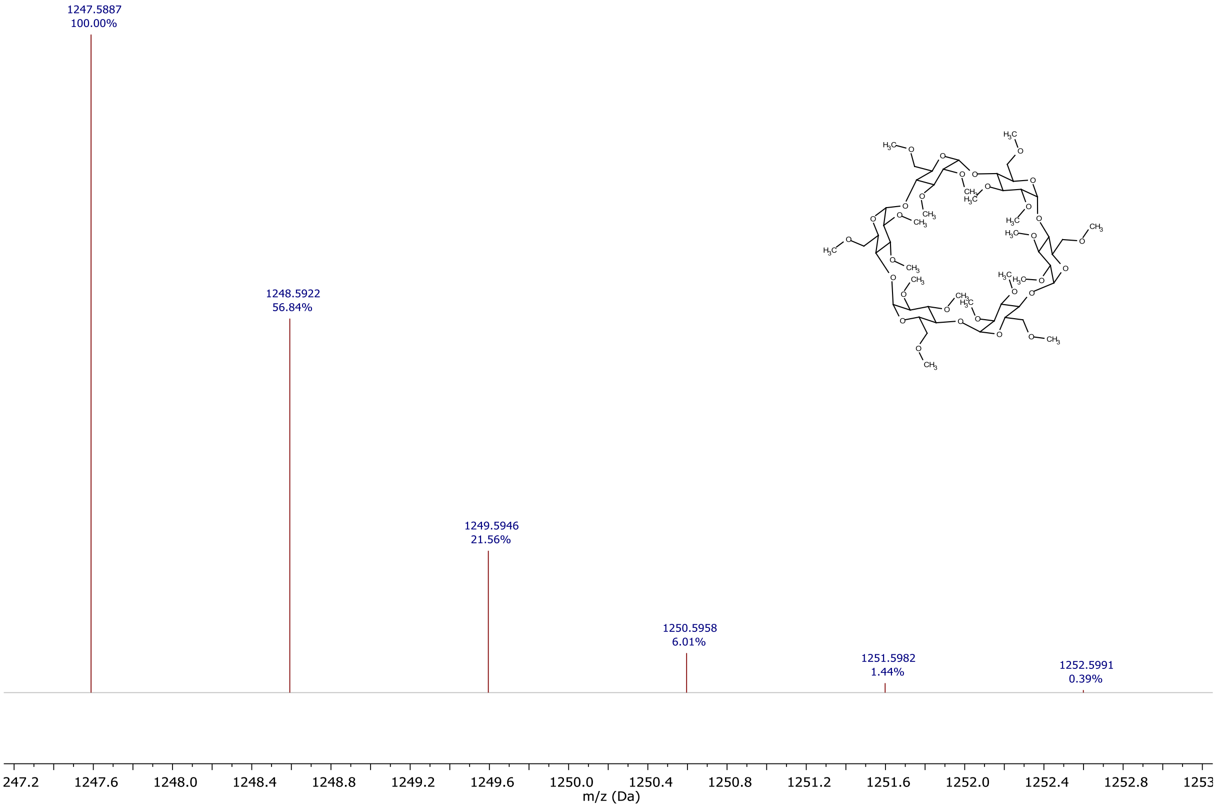

High resolution mass spectra of hexakis(2,3,6-tri-*O*-methyl)-α-cyclodextrin.

S3. X-Ray crystal structure of hexakis(2,3,6-tri-*O*-methyl)-α-cyclodextrin crystallized from trichlorofluoromethane

For crystallographic analysis hexakis(2,3,6-tri-*O*-methyl)-α-cyclodextrin (15 mg) is dissolved in 1 mL trichlorofluoromethane. After partly evaporation (ca. 0.3 mL) of trichlorofluoromethane at room temperature, clear colorless crystals of crystallographic quality are obtained within less than one hour and investigated by X-ray crystal structure analysis.

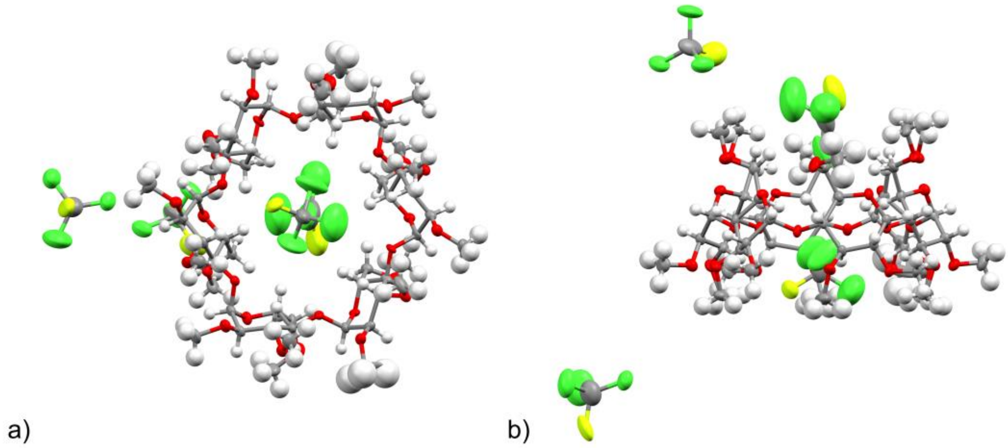

Figure S1: Thermal ellipsoid molecular structure of crystallized complex of hexakis(2,3,6-tri-*O*-methyl)-α-cyclodextrin with trichlorofluoromethane determined by X-ray crystal structure analysis. a) Plan-view showing the cyclodextrins's secondary side, b) side-view of the crystallized inclusion complex. Color code: C gray, O red, Cl green, F yellow, H white.

The X-ray analysis of the crystals reveals a tetragonal $I4_1$ space group of the crystallized inclusion complex, with four trichlorofluoromethane molecules being enclosed per hexakis(2,3,6-tri-*O*-methyl)-α-cyclodextrin molecule (**Figure S1** and **S2**). Thereby, two of the CFC-11 guests are positioned in the interstitial cavities of the adjacent cyclodextrins, one CFC-11 molecule is halfway inside the cyclodextrin cavity at its primary side and the remaining CFC-11 guest is located fully inside the hexakis(2,3,6-tri-*O*-methyl)-α-cyclodextrin cavity.

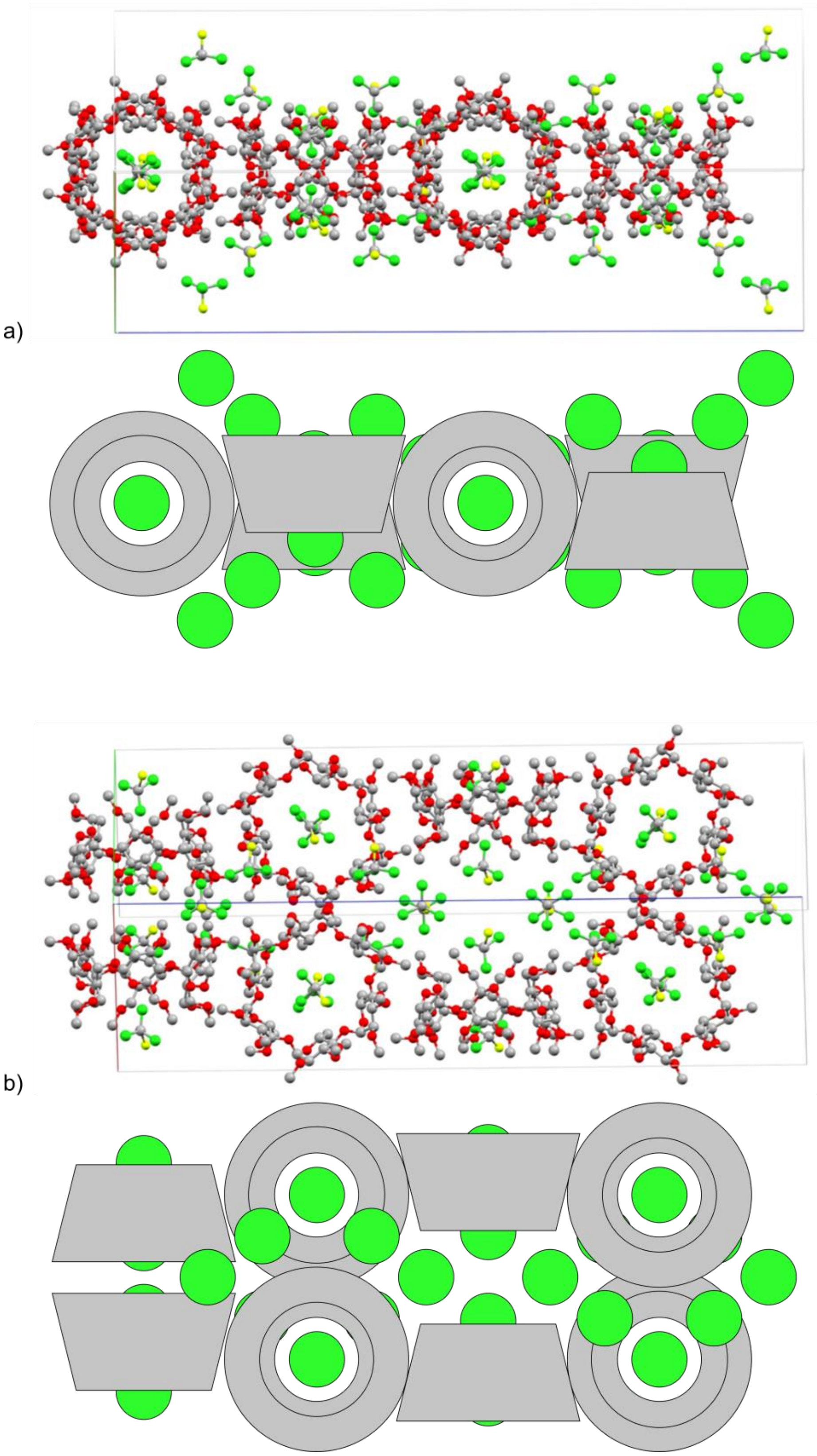

Figure S2: Packing of the hexakis(2,3,6-tri-*O*-methyl)-α-cyclodextrin unit with the encapsulated trichlorofluoromethane determined by X-ray crystal structure analysis. Hydrogen atoms are omitted for clarity. Color code: C gray, O red, Cl green, F yellow.

In this configuration, the cavities of the hexakis(2,3,6-tri-*O*-methyl)-α-cyclodextrin molecules form channels through the crystal lattice. The hexakis(2,3,6-tri-*O*-methyl)-α-cyclodextrin

molecules are oriented with a right turn rotation of 90° from one molecule to another along the c-axis and with a 180° rotation in the a,b-direction of the crystal lattice.

Table S1. Crystal data and structure refinement for hexakis(2,3,6-tri-*O*-methyl)-α-cyclodextrin crystallized from trichlorofluoromethane (CCDC-1841752).

Empirical formula (with solvates)	$C_{54}H_{96}O_{30}$, 4(CCl_3F)
Formula weight (incl. solvates)	1774.74
Temperature / K	120
Crystal system	tetragonal
Space group	I 4_1
$a = b$ / Å	17.81508(19)
c / Å	54.0850(8)
Volume/ Å^3	17165.3(4)
Z	8
ρ_{calc} g / cm^3	1.373
μ / mm^{-1}	0.468
F(000)	7392
Crystal size / mm^3	0.17 x 0.37 x 0.63
Radiation	Mo-Kα
2Θ range for data collection	1.8 to 28.4°
Indx range	$-23 \leq h \leq 23$, $-23 \leq k \leq 23$, $-67 \leq l \leq 71$
Reflections collected	289547
Independent reflections	21068 (R_{int} = 0.0503)
Data / restraints / parameters	17237 (|F|/σ(F) > 4.0)
Goodness-of-fit	1.039
Final R indexes [$I \geq 2\sigma(I)$]	R_1 = 0.0761
Final R indexes [all data]	R_1 = 0.095, wR_2 = 0.2374
Largest diff. peak/hole / e Å^{-3}	0.94/–0.97

S4. X-Ray crystal Structure of hexakis(2,3,6-tri-*O*-methyl)-α-cyclodextrin crystallized from a mixture of dichloromethane and trichlorofluoromethane (95:5)

For crystallographic analysis hexakis(2,3,6-tri-*O*-methyl)-α-cyclodextrin (15 mg) is dissolved in 1 mL of a trichlorofluoromethane-dichloromethane mixture with a ratio of 5:95. After partly evaporation of the binary solvent mixture, clear colorless crystals can be obtained within less than one hour and are investigated by X-ray crystal structure analysis.

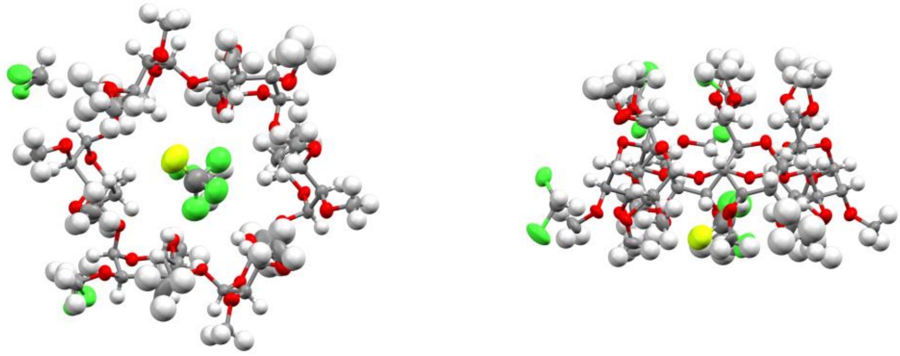

a) b)

Figure S3: Thermal ellipsoid molecular structure of the crystallized complex of hexakis(2,3,6-tri-*O*-methyl)-α-cyclodextrin with dichloromethane and trichlorofluoromethane determined by X-ray crystal structure analysis. a) Plan-view showing the cyclodextrin's secondary side. b) Side-view of the crystallized inclusion complex. Color code: C gray, O red, Cl green, F yellow, H white.

The X-ray analysis of the crystals reveals an orthorhombic complex with a $P\ 2_1 2_1 2_1$ space group. The X-ray analysis shows a complex with three dichloromethane molecules and one trichlorofluoromethane molecule being enclosed per hexakis(2,3,6-tri-*O*-methyl)-α-cyclodextrin molecule (**Figure S3** and **S4**). Thereby, two of the DCM guests are positioned in the interstitial cavities of the adjacent cyclodextrins, the last DCM molecule is positioned inside the cyclodextrin cavity at its primary side and the CFC-11 guest is located fully inside the hexakis(2,3,6-tri-*O*-methyl)-α-cyclodextrin cavity at its secondary side. Furthermore, cyclodextrin molecules form layers arranged in the a-direction of the lattice. In b-direction of the crystal lattice a channel building through the cavities of the cyclodextrin molecules can be observed.

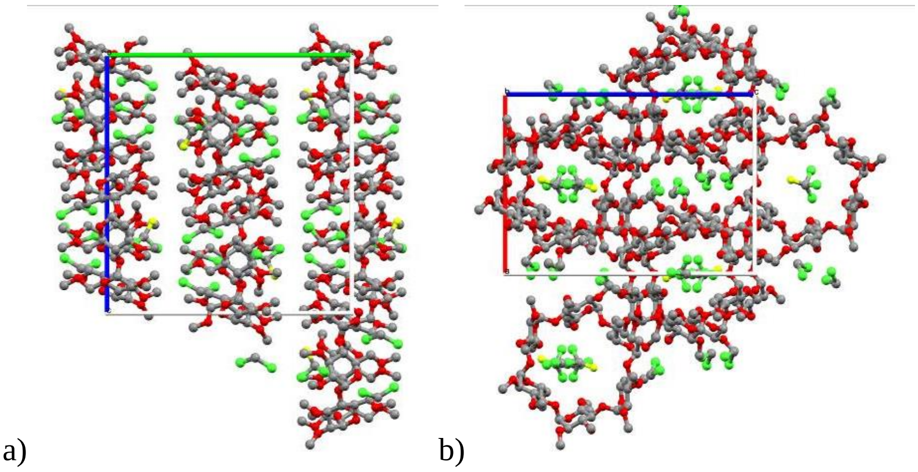

a) b)

Figure S4: Packing of the hexakis(2,3,6-tri-*O*-methyl)-α-cyclodextrin unit with the encapsulated trichlorofluoromethane and dichloromethane determined by X-ray crystal structure analysis, with a) view from a-direction and b) view from b-direction. Hydrogen atoms are omitted for clarity. Color code: C gray, O red, Cl green, F yellow.

The encapsulation of exclusively CFC-11 within the inner cavity of hexakis(2,3,6-tri-*O*-methyl)-α-cyclodextrin highly exceeds the normal statistical distribution of the whole complex and especially of the cyclodextrin inner cavity five-fold and twenty-fold, respectively, thus, proving the high adsorption selectivity of hexakis(2,3,6-tri-*O*-methyl)-α-cyclodextrin to CFC-11.

Table S2. Crystal data and structure refinement for hexakis(2,3,6-tri-*O*-methyl)-α-cyclodextrin crystallized from mixture of dichloromethane and trichlorofluoromethane (95:5) (CCDC-1841753).

Empirical formula (with solvates)	$C_{54}H_{96}O_{30}$, CCl_3F, $3(CH_2Cl_2)$
Formula weight (incl. solvates)	1617.44
Temperature / K	120
Crystal system	orthorhombic
Space group	P $2_12_12_1$
a / Å	15.7397(4)
b / Å	21.7162(7)
c / Å	22.6475(8)
Volume/ $Å^3$	7741.1(4) (4)
Z	4
ρ_{calc} g / cm^3	1.388
μ / mm^{-1}	0.41
F(000)	3408
Crystal size / mm^3	0.14 x 0.44 x 0.55
Radiation	Mo-K*α*
2*Θ* range for data collection	2 to 28°

Indx range	$-20 \le h \le 19$, $-28 \le k \le 28$, $-29 \le l \le 29$
Reflections collected	67315
Independent reflections	18481 (R_{int} = 0.0486)
Data / restraints / parameters	11363 ($\lvert F\rvert/\sigma(F) > 4.0$)
Goodness-of-fit	1.026
Final R indexes [$I \geq 2\sigma(I)$]	R_1 = 0.0979
Final R indexes [all data]	R_1 = 0.1485, wR_2 = 0.2994
Largest diff. peak/hole / e Å^{-3}	0.83/–0.71

S5. TGA and TGA-MS experiments of hexakis(2,3,6-tri-*O*-methyl)-α-cyclodextrin inclusion complex with trichlorofluoromethane

TGA measurements reveal a thermal stability of the inclusion complex up to a temperature of 85 °C (**Figure S5**). This temperature marks the onset of a stepwise guest release and is 60 °C above the boiling point of pure CFC-11 (23.6 °C).

Incorporating TGA-MS analyses reveal that exclusively CFC-11 is released in the temperature range between 85 °C and 195 °C, with hexakis(2,3,6-tri-*O*-methyl)-α-cyclodextrin being stable up to a temperature of approx. 270 °C. The occurring weight loss of 28 wt% up to a temperature of 195 °C perfectly fits the complex ratio of 1:4 of hexakis(2,3,6-tri-*O*-methyl)-α-cyclodextrin and CFC-11 as determined by the X-ray analyses and corresponds to 93% of maximum CFC-11 capacity in the lattice.

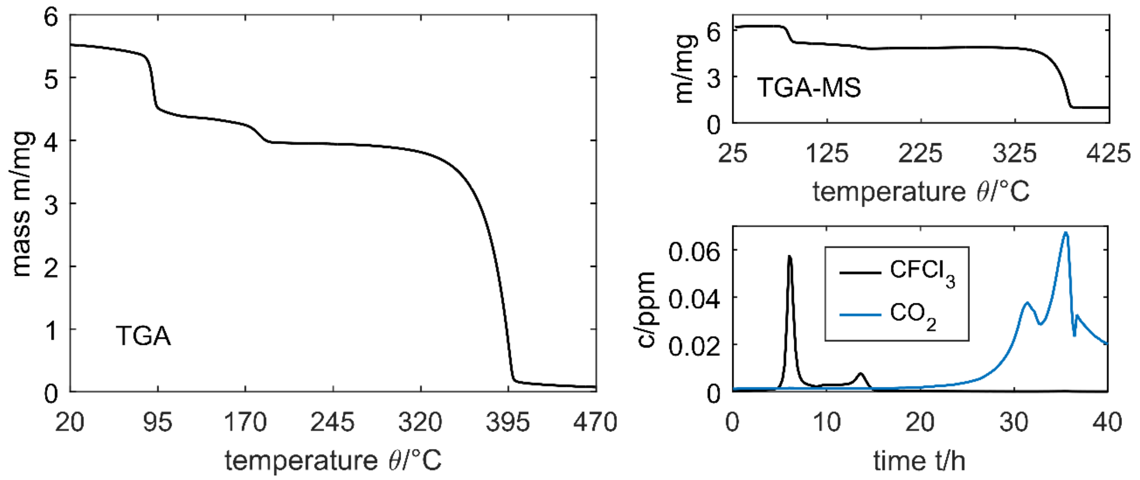

Figure S5: TGA measurement of the trichlorofluoromethane in hexakis(2,3,6-tri-*O*-methyl)-α-cyclodextrin supramolecular complex (left) and TGA-MS measurements of the complex with corresponding TGA data (right top) and mass data (right bottom).

Further TGA measurements demonstrate the thermal stability of the complex under ambient conditions. Here, the complex of CFC-11 and hexakis(2,3,6-tri-*O*-methyl)-α-cyclodextrin (10.1032 mg) is isothermally measured at 25 °C while exposition to a nitrogen flow of

20 mL·min^{-1} in the TGA apparatus for 12 hours. During this measurement, a weight loss of only 3.5 wt% (0.3569 mg) could be observed (**Figure S6**) and therefore proves the high stability of the complex.

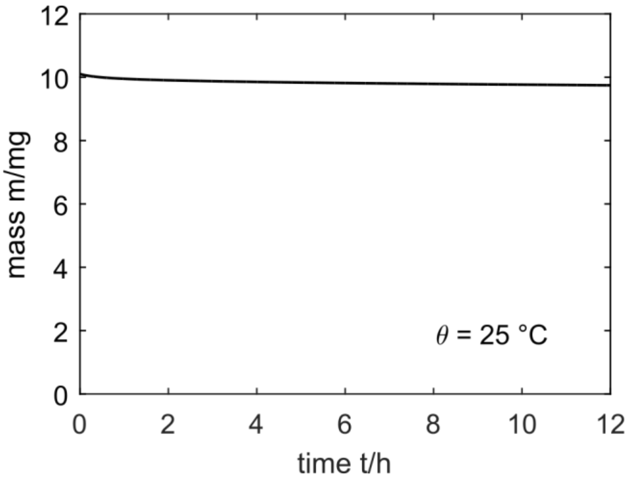

Figure S6: Long term stability thermogravimetric measurement at isothermal conditions (25 °C) for 12 hours with nitrogen stream of 20 mL·min^{-1}.

S6. Gas mixing unit

In order to investigate the applicability of the inclusion complex formation of gaseous CFC-11 with hexakis(2,3,6-tri-*O*-methyl)-α-cyclodextrin, a gas mixing unity is applied (**cf. Figure S7**). In this gas mixing unit, an inert nitrogen gas flow is divided into two separate streams, which are both controlled by an individual mass flow controller (MFC) (Brooks Instrument, Model 5050S). While the first stream remains unchanged in temperature and composition and is used for dilution purposes, the second stream is led through an interchangeable and temperature controlled CFC-11-reservoir. The temperature of the CFC-11 reservoir is set to 288.2±0.01 K which results in a concentration of 740950 ppm for the fully saturated nitrogen gas stream. The final CFC-11 concentration is adjusted by recombining the saturated gas flow 2 with the pure inert gas flow 1 in proportionate measures. The mixed gas of defined CFC-11 concentration is then led through an interchangeable test-device before it passes a condenser, that is cooled down to a temperature of approx. 180 K using a cooling bath of dry ice and acetone, and extracts any remaining CFC-11 from the gas stream.

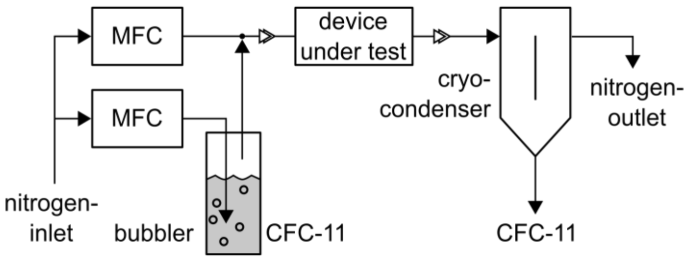

Figure S7: Schematic illustration of the gas mixing unit.

S7. Adsorption of airborne CFC-11 by hexakis(2,3,6-tri-*O*-methyl)-α-cyclodextrin

To perform adsorption experiments on a macroscopic scale, an experimental setup is built, which is based on a packed-bed adsorption-tube connected to the gas mixing unit (**Figure S8**). For the adsorption, a glass tube that features an inner diameter of 12 mm is filled with 6.45 g hexakis(2,3,6-tri-*O*-methyl)-α-cyclodextrin, sealed with glass frits and connected to the previously described gas mixing unit. The adsoprtion experiments are performed at a temperature of 293.2±0.1 K. The overall gas flow is set to 60 mL·min^{-1}.

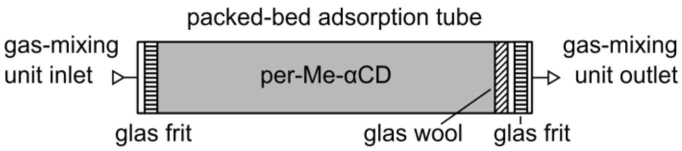

Figure S8: Schematic illustration of the hexakis(2,3,6-tri-*O*-methyl)-α-cyclodextrin filled packed-bed adsorption-tube.

The adsorption tube is exposed to a gas flow with 50 vol% of CFC-11. In order to quantify the amount of CFC-11 with hexakis(2,3,6-tri-*O*-methyl)-α-cyclodextrin for adsorption purposes, the adsorption-tube is weighted prior and subsequently to the CFC-11 exposure. Here, a weight increase of an average of 20.0 wt% can be found (**Figure S9** and **Table S3**).
Furthermore, the adsorption can be completely reversed through the heating of the adsorption-tube up to a temperature of 85 °C while purging with a pure nitrogen stream of 60 mL·min^{-1}. The average residual weight increase of the adsorption-tube after the desorption experiments is found to be <0.1 wt%.
To prove the stability of the adsorption of CFC-11 on hexakis(2,3,6-tri-*O*-methyl)-α-cyclodextrin, the adsorption-tube was exprosed to a nitrogen flow of 60 mL·min^{-1} over a period of 12 hours after the adsorption experiment. While this time period a weight decrease of an average of 5 wt% could be observed, which lies in a very good agreement to the TGA experiments (3 wt%, **Figure S6**).

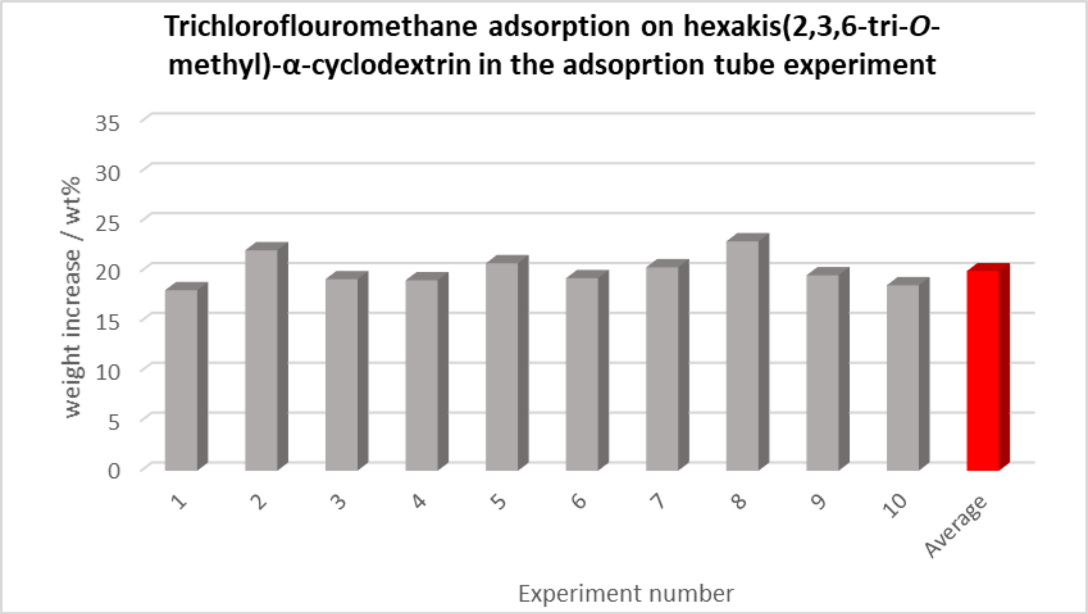

Figure S9: Trichloroflouromethane adsorption on hexakis(2,3,6-tri-*O*-methyl)-α-cyclodextrin in the adsorption tube experiment.

Table S3: Trichloroflouromethane adsorption on hexakis(2,3,6-tri-*O*-methyl)-α-cyclodextrin in the adsorption tube experiment.

**Experiment number**	**Weight increase of the adsorption tube after adsorption of CFC-11 / wt%**	**Residual weight increase of the adsorption tube after desorption of CFC-11 / wt%**
**1**	18.1	<0.1
**2**	22.1	0.1
**3**	19.2	<0.1
**4**	19.1	<0.1
**5**	20.8	0.1
**6**	19.3	<0.1
**7**	20.4	<0.1
**8**	23.0	<0.1
**9**	19.6	<0.1
**10**	18.6	0.1
**Average**	20.0	<0.1

S8. Fabrication and principle of the planar Bragg grating sensor

Bragg gratings (BGs) represent a periodic perturbation of the refractive index within a light guiding medium. Most commonly, Bragg gratings are fabricated in optical waveguides, such as optical fibers or planar waveguides. Here, the waveguide features a higher refractive index as the surrounding cladding material, while most commonly, the index perturbation of the

Bragg grating features a slightly higher refractive index as compared to the waveguide core (**Figure S10**).

Bragg gratings have the ability to act as a dielectric mirror for a defined wavelength according to the Bragg condition (**Equation S1**):

$$m \cdot \lambda_B = 2 \cdot n_{eff} \cdot \Lambda \quad \text{(S1)}$$

with the reflected wavelength (referred to as the Bragg wavelength) λ_B the effective refractive index of the respective guided mode interacting with the Bragg grating n_{eff}, the perturbation period of the refractive index variation within the waveguide Λ, and the order of reflection m (which is a natural number).[S3]

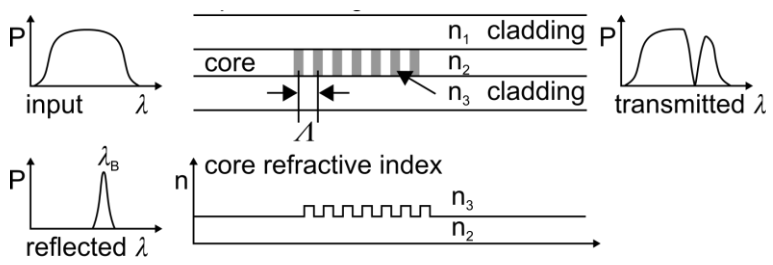

Figure S10: Basic principle and design of waveguide Bragg gratings.

Therefore, if a broad light-spectrum is traveling through the waveguide a small part of the light, which matches the Bragg condition, is reflected by the Bragg grating, while complementarily, the rest of the spectrum is transmitted through the Bragg grating.

Since the reflected Bragg wavelength is depending on the effective refractive index and the grating period, any variable that influences one or both of these parameters leads to a variation of the reflected Bragg wavelength and can accordingly be detected and monitored with an appropriate interrogation equipment.

Prominent examples of influencing variables that are well detectable with Bragg gratings are temperature and deformation. Both lead to a, either temperature or stress induces alteration of the refractive index as well as an alteration of the grating period, either through thermal expansion or tensile strain.[S4]

A further scope of application can be opened as soon as the light guiding through the waveguide is able to interact with a varying surrounding medium. By partly omitting the cladding at a Bragg grating, a small portion of the guided mode, the so-called evanescent field, interacts with the surrounding media, whose refractive index therefore incorporates the effective refractive index of this respective guided mode and, thus, influences the reflected Bragg wavelength (**Figure S11**).

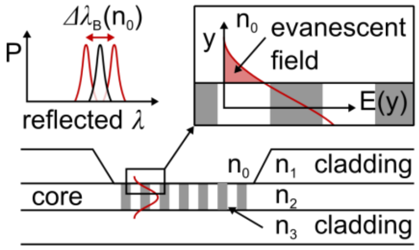

Figure S11: Schematic illustration of a Bragg grating based refractive index sensor.

By this measure, changes in the surrounding media in the vicinity of the Bragg grating, that incorporate a refractive index alteration can be detected and monitored with an appropriate interrogation equipment.[S5,S6]

The here applied planar Bragg grating (PBG) sensor is based on a multilayer structure of silica on a silicon wafer. During the epitaxial growth of the silica using flame hydrolysis, intermediately, germanium is added to the process resulting in a sandwich-like stacked structure of silica, Ge-doped silica and silica on top of the wafer. Due to the doping of the middle layer, this layer becomes photo-sensible, which allows a simultaneous inscription of both, waveguide and Bragg grating using a direct writing technique which is based on UV-laser modification of the materials refractive index.[S6,S7]

By this measure, multiple waveguide Bragg gratings of varying grating period are inscribed into the middle layer, which is vertically enclosed by a silica cladding and horizontally enclosed by a non-modified Ge-doped silica cladding. Each Bragg grating leads to a defined Bragg reflection peak according to the Bragg condition (**Equation S1**).

In order to guarantee an evanescent field interaction of the guided mode with the surrounding media of the Bragg gratings, the silica top-cladding above two Bragg gratings is subsequently removed using wet etching technique that results in an ellipse-shaped sensing window. In order to further enhance the sensitivity, the sensor surface is covered with a 50 nm thin high-refractive index film of titan dioxide which causes a slight pull of the guided mode towards the surface and hence in a more pronounced evanescent field interaction with the surrounding medium. Any remaining Bragg gratings, that are located between the sensing window and the coupling facet of the inscribed waveguide remain buried under the top-cladding and are thus uninfluenced by the high-refractive index film and the surrounding media and thus serve as a reference for intrinsically interfering influences, such as temperature changes. A detailed description of the planar Bragg grating fabrication process can be found by the authors and others elsewhere.[S5,S6]

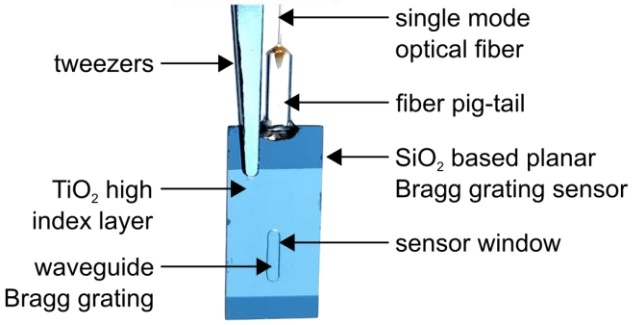

Figure S12: Photography of the applied planar Bragg grating based refractive index sensor.

In order to utilize the sensor chip, a single mode optical fiber pig-tail (a pre-fabricated fiber that comprises an FC/APC connector) is butt-coupled and bonded to the coupling-facet of the sensor chip using a UV-curable adhesive. In the here presented experiments, the ready-to-use sensor chip (type S7b by Stratophase Ltd., UK) is connected to a combined source and detector interrogation system (sm125 by micron optics, GA., USA) that operates in the telecom wavelength range at a resolution of 1 pm and allows a detection and tracking of the reflected Bragg wavelengths at a sampling rate of 2 Hz.

The characteristic of Bragg gratings allow an indirect detection and monitoring of the surrounding refractive index, furthermore allows their application in the field of bio- and chemical sensing as utilized in this work.

S9. PBG sensor coating and layer thickness determination

The PBG sensor is coated with hexakis(2,3,6-tri-*O*-methyl)-α-cyclodextrin using a dip coating unit (WPTL5-0.01 from MTI Corporation, CA., USA). For this purpose, the hexakis(2,3,6-tri-*O*-methyl)-α-cyclodextrin is dissolved in tetrahydrofuran ($c = 50\ g \cdot L^{-1}$). By choosing a withdrawal speed of 150 $mm \cdot min^{-1}$, a functional receptor layer thickness of approximately 150 nm is achieved as determined using a Stylus Surface Profiler (DektakXT by Bruker Corporation, MA., USA). This measure ensures an appropriate overlap of the evanescent field with the applied affinity material. The dwell time and entry speed have no appreciable effect on the sensing behavior and are therefore chosen to 3 s and 150 $mm \cdot min^{-1}$, respectively. Therefore, inclusion complex formation of the host cyclodextrin with an appropriate CFC-11 guest molecule results in a slight alteration of the functional layer's refractive index and consequently in a distinct and analyzable shift of the reflected Bragg wavelength.

S10. Detection of gaseous CFC-11 using a hexakis(2,3,6-tri-*O*-methyl)-α-cyclodextrin coated planar Bragg grating

For the detection of gaseous CFC-11, a planar Bragg grating sensor is applied which is coated with a 150 nm thin layer of hexakis(2,3,6-tri-*O*-methyl)-α-cyclodextrin. In the experimental setup, the CFC-11 sensitized sensor as well as an identical but uncoated sensor are placed in a series of sealed gas-flow chambers. The flow chambers are connected to the previously described gas mixing unit and tempered to 308.1±0.1 K. The temperature of the gas-flow chamber is chosen to 15 K above the analyte temperature in order to prevent unspecific condensation of the CFC-11 on the sensor surface. The PBG sensors themselves are connected to the previously mentioned optical interrogation system (**Figure S13**).

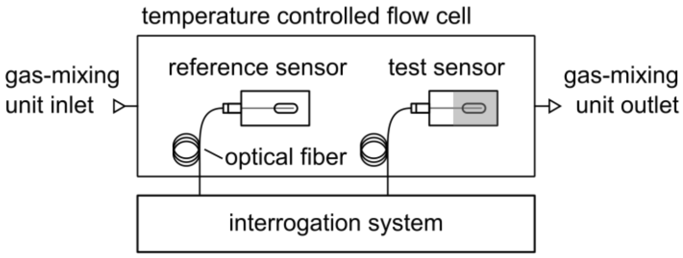

Figure S13: Experimental setup for the detection of gaseous CFC-11 using a hexakis(2,3,6-tri-*O*-methyl)-α-cyclodextrin coated planar Bragg grating sensor.

In order to ensure stable initial conditions, approx. 18 hours prior to the measurements, the PBG sensors are tempered and exposed to a constant nitrogen flow of 200 mL·min^{-1}. During the measurement, the PBG sensors are exposed to a series of ascending CFC-11 concentrations with intermediate purging of pure nitrogen. The applied concentrations are 1%, 2%, 5%, and 10%. While the sensor signal of the hexakis(2,3,6-tri-*O*-methyl)-α-cyclodextrin coated PBG sensor reveals a distinct deflection to CFC-11 application, the uncoated control sensor shows a minor response only to higher CFC-11 concentrations (**Figure S14**). Exemplarily, for an applied CFC-11 concentration of 10%, the uncoated PBG sensor exhibits a signal deflection of 1.8 pm, while the sensitized sensors exhibits a signal deflection of 82 pm, which represents a 45-fold signal increase that proves the suitability of hexakis(2,3,6-tri-*O*-methyl)-α-cyclodextrin as an affinity material for the chemical sensing of CFC-11.

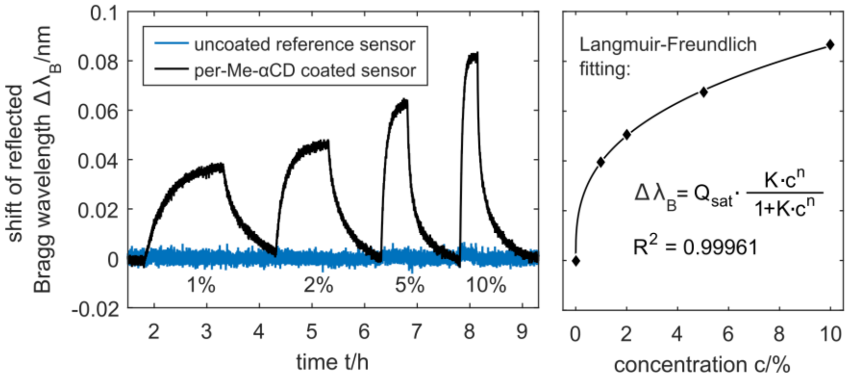

Figure S14: Response of the hexakis(2,3,6-tri-*O*-methyl)-α-cyclodextrin coated and uncoated PBG sensor to CFC-11 application (left) and the corresponding Langmuir-Freundlich fit (right)

The coated, hence sensitized sensor exhibits an immediate jet damped saturating signal response to CFC-11 application, where the sensor signal increases with increasing CFC-11 concentration and the intermediate purge of pure nitrogen leads to a signal recovery towards the initial state. The dependency of the sensors total signal response on the respective applied CFC-11 concentration shows a Langmuir-Freundlich isotherm ($R^2 = 0.999$),[S8] which can be attributed to a growing occupation of the near-surface cyclodextrin hosts with increasing CFC-11 concentration and a diffusion of the CFC-11 guests into the cyclodextrin layer at higher concentrations. This concentration-depended signal increase of the coated Bragg grating sensor can be used for sensor application purposes, in order to detect small CFC-11 concentrations out of waste gas stream or in industrial facilities.

S11. Crystallization of the surface coating on the Bragg grating sensor

For CFC-11 concentrations above 35 vol% a spontaneous, clearly visible crystallization of the amorphous hexakis(2,3,6-tri-*O*-methyl)-α-cyclodextrin sensitization layer of the planar Bragg grating sensor occurs, which leads to an instantaneous signal drop-off of the sensor. Thereby, the signal drop-off can be attributed to firstly an increasing refractive index of the hexakis(2,3,6-tri-*O*-methyl)-α-cyclodextrin layer due to the deposition of CFC-11 that results in a cumulative pull of the guided mode towards the surface of the waveguide and secondly to scattering losses due to the crystallization caused structural discontinuity of the sensor surface. The crystallization occurs within few seconds after exposition to CFC-11 containing gas stream.

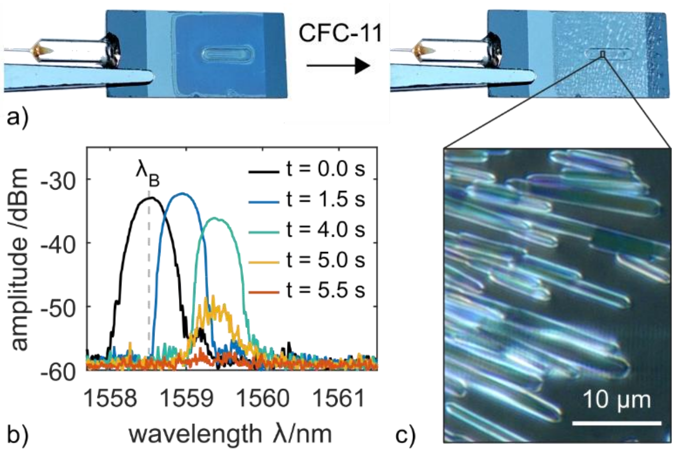

Figure S15: a) Photography of the functionalized planar Bragg grating sensor before (left) and after (right) exposure to CFC-11. b) Shift of the reflected Bragg wave length with signal drop-off, due to the crystallization. c) Microscopic image of the crystalized sensor surface (**2** @ **1**).

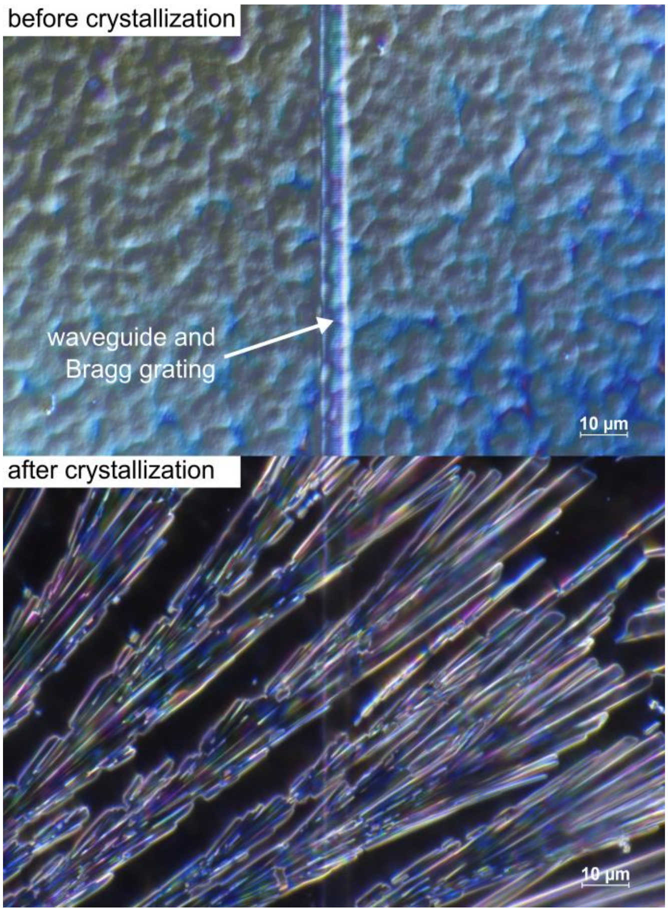

Figure S16: Microscopic images of the coated amorphous sensor surface (top) and crystalized sensor surface after the exposition to CFC-11 (bottom).

S12. References

[S1] G. M. Sheldrick, *Acta Crystallogr., Sect. A: Found. Crystallogr.* **2008**, *64*, 112.

[S2] a) Y. Akae, Y. Koyama, H. Sogawa, Y. Hayashi, S. Kawauchi, S. Kuwata, T. Takata, *Chem. - Eur. J.* **2016**, *22*, 5335; b) J. Boger, R. J. Corcoran, J.-M. Lehn, *Helv. Chim. Acta* **1978**, *61*, 2190; c) B. Casu, M. Reggiani, G. G. Gallo, A. Vigevani, *Tetrahedron* **1968**, *24*, 803; d) G. Schomburg, A. Deege, H. Hinrichs, E. Hübinger, H. Husmann, *J. High Resol. Chromatogr.* **1992**, *15*, 579.

[S3] a) A. Othonos, *Rev. Sci. Instrum.* **1997**, *68*, 4309; b) K. O. Hill, G. Meltz, *J. Lightwave Technol.* **1997**, *15*, 1263; c) A. Cusano, A. Cutolo, J. Albert, *Fiber Bragg Grating Sensors. Recent Advancements, Industrial Applications and Market Exploitation*, Bentham Science Publishers, **2012**.

[S4] a) M. Girschikofsky, M. Rosenberger, M. Förthner, M. Rommel, L. Frey, R. Hellmann, *Sensors* **2017**, *17*, 2459; b) M. Rosenberger, S. Hessler, S. Belle, B. Schmauss, R. Hellmann, *Opt. Express* **2014**, *22*, 5483; c) W. Lu, G. Cai, W. Liu, W. Xing (Eds.) *Lecture Notes in Electrical Engineering*, Springer Berlin Heidelberg, Berlin, Heidelberg, **2013**; d) B. Lee, *Opt. Fiber Technol.* **2003**, *9*, 57; e) Y.J. Rao, *Optics and Lasers in Engineering* **1999**, *31*, 297.

[S5] S. Scheurich, S. Belle, R. Hellmann, S. So, I. J. G. Sparrow, G. Emmerson in *SPIE Proceedings* (Eds.: F. Baldini, J. Homola, R. A. Lieberman), SPIE, **2009**, 73561B.

[S6] I. J. G. Sparrow, P. G. R. Smith, G. D. Emmerson, S. P. Watts, C. Riziotis, *J. Sens.* **2009**, *2009*, 1.

[S7] G. D. Emmerson, S. P. Watts, C.B.E. Gawith, V. Albanis, M. Ibsen, R. B. Williams, P.G.R. Smith, *Electron. Lett.* **2002**, *38*, 1531.

[S8] a) O. Redlich, D. L. Peterson, *J. Phys. Chem.* **1959**, *63*, 1024; b) I. Langmuir, *J. Am. Chem. Soc.* **1918**, *40*, 1361.
